# Type I Sourdough Preservation Strategies and the Contribution of Microbial Biological Resource Centers to Biodiversity Protection: A Narrative Review

**DOI:** 10.3390/foods14152624

**Published:** 2025-07-26

**Authors:** Roberta Coronas, Angela Bianco, Anna Maria Laura Sanna, Giacomo Zara, Marilena Budroni

**Affiliations:** Department of Agricultural Sciences, University of Sassari, 07100 Sassari, Italy; r.coronas3@phd.uniss.it (R.C.); abianco@uniss.it (A.B.); a.sanna82@studenti.uniss.it (A.M.L.S.); mbudroni1@uniss.it (M.B.)

**Keywords:** sourdough, microbiome preservation, mBRC, biodiversity protection, microbial community

## Abstract

Traditional type I sourdoughs are being rediscovered and increasingly used in artisanal and industrial bakeries due to the unique taste and texture, potential health benefits, and longer shelf life they confer on to baked products. These unique properties are attributed to the diverse microbial communities of sourdough, comprising both yeasts and bacteria. The traditional preservation method for type I sourdough (i.e., continuous backslopping) may lead, over time, to taxonomic and functional rearrangements of its microbial communities. Consequently, significant deviations in the characteristics of baked products can occur. In this context, this review aims to summarize the recent literature on the long-term preservation and maintenance strategies for type I sourdough and highlight the essential role that microbial biological resource centers (mBRCs) could play in the preservation and sharing of sourdough microbiomes. Specifically, the identification of appropriate preservation methods, implementation of well-defined access and benefit-sharing protocols, and development of microbiome-specific datasets, should be encouraged within the context of mBRCs. These infrastructures are expected to play a pivotal role in preserving the microbiota of fermented foods, serving as a crucial element for innovation and the safeguarding of traditional foods and culinary heritage.

## 1. Introduction

Bread-making is an ancient tradition that dates back almost 14,400 years ago [1,2]. Bread is closely linked to human subsistence and embraces knowledge, technological processes, society, and religion [3]. Historically, bread was leavened by a natural starter originating from the spontaneous fermentation of heterogeneous mixtures of flour and water by naturally occurring bacteria and yeasts [4]. This starter, named sourdough because of its low pH, represents one of the most ancient and long-lasting examples of a natural fermentation starter in food processing and continues to be a subject of extensive research [5,6]. Traditional sourdough, currently classified as type I sourdough, contains high populations of lactic acid bacteria (LAB) and yeasts (approximately 9 and 7 log CFU/g, respectively) in a ratio ranging from 10:1 to 100:1 [7]. Each traditional sourdough starter possesses a complex microbiome, which typically originates from the assembly of microbial communities in the environment, flour, and water, under the selective pressures exerted by backslopping techniques and specific technological parameters, such as temperature, percentage of hydration, time, etc. [8]. Thus, the microbiome of each type I sourdough is unique, and effective methods for its long-term preservation are needed to ensure bread-fermentation reproducibility, preserve microbial biodiversity, and ensure a source of microbial cultures for sustainable innovation in food technology. Indeed, the traditional method for maintaining type I sourdough, namely continuous backslopping, can lead to taxonomic and functional shifts in the resident microbiota over time. These alterations may result from processing conditions, environmental fluctuations, or random events, such as bacteriophage infections. Such dynamics can ultimately affect the sensory and technological properties of the baked products. Therefore, the long-term preservation of the microbiota of a mature sourdough may represent a valuable strategy to restore or maintain the original characteristics of the final bread, particularly following perturbations or extreme events. The preserved microbiota could function analogously to the bakery environment, often referred to as the “house microbiota” [9], by contributing to the re-establishment of sourdough microbial communities. Notably, several studies have demonstrated that the propagation environment (e.g., different bakeries or bakery vs. laboratory conditions) plays a key role in shaping both the species- and strains-level composition of the sourdough microbiota, regardless of flour type or processing parameters [10].

In this context, this review illustrates the main strategies for the maintenance and preservation of sourdough microbiota, both at the community level and as individual strains. The de novo design of reconstructed microbial communities as a method of sourdough reproduction and preservation has also been analyzed. Finally, this review covers the role of microbial collections in certifying the quality of preserved sourdough microbiota and its associated data, with reference to the regulatory framework for biodiversity preservation. To assess these aims, the bibliographic databases Scopus and Google Scholar were consulted using the following queries: “sourdough drying” AND/OR “freeze-dried sourdough” AND/OR “spray-dried sourdough” AND/OR “microbiome preservation” AND/OR “Biobanking”.

## 2. Economic Relevance of Sourdough-Derived Products and Innovation Need

**Figure 1:**
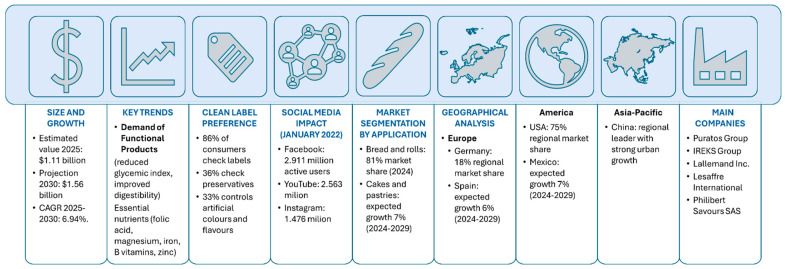
Global sourdough market analysis overview showing key metrics from 2025−2030 projections, market trends, consumer preferences, geographical distribution, and major industry players. Summary of data from [11].

The sourdough market is growing strongly, with projected sales of USD 1.11 billion in 2025, and is expected to reach USD 1.56 billion by 2030 (Figure 1).

**Figure 2:**
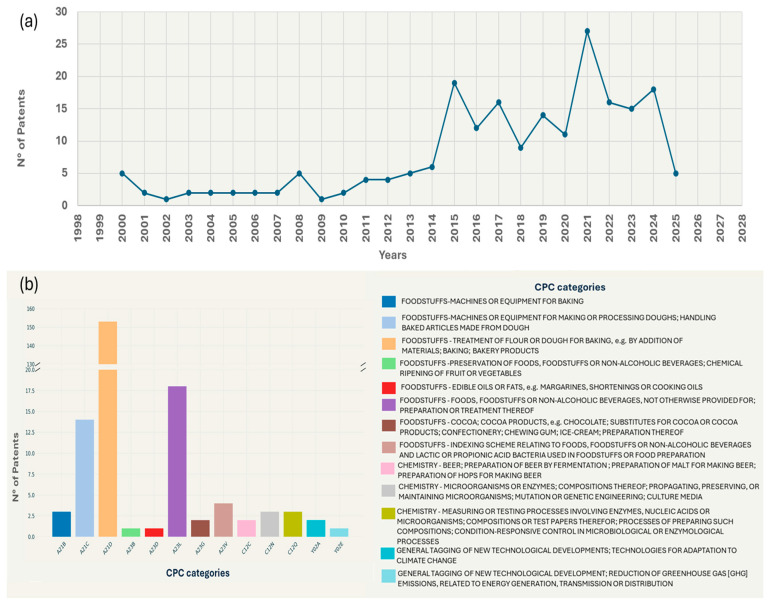
Survey of sourdough-related patents over the observation period (2000−2025): (**a**) number of patents published per year and (**b**) distribution of patents across Cooperative Patent Classification (CPC) categories.

The increasing demand for high-quality sourdough-derived products is driven by the demand for gluten-free, organic, and specialty flavored products, pseudocereal flours, and more sustainable food products. Europe is expected to dominate the global sourdough market by 2027, with Germany as the main market, followed by France, Italy, and Spain. Automated production systems ensure large-scale production, thereby expanding distribution to consumers who recognize the nutritional properties of sourdough-derived goods [11]. However, the high costs required to maintain the stability and biodiversity of sourdough microbiota render such products economically uncompetitive when compared with those made with baker’s yeast [12]. Thus, further global market expansion of sourdough-derived products requires innovations in both product and production processes. Accordingly, between 2000 and 2025, 207 patents with the term “SOURDOUGH” in the title and/or abstract were identified on the Espacenet database [13] (Figure 2).

**Figure 3:**
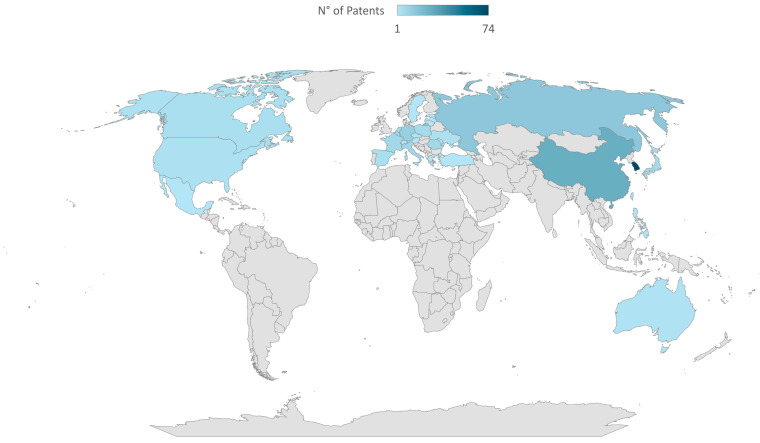
Geographical distribution of applicant affiliations for sourdough-related patents (2000−2025).

Overall, the main innovations include the optimization of fermentation processes (patents DE19938278A1, US6508164B1) [14,15], the use of new microbial strains (patents FR2781811A1, EP1110458A1) [16,17], the development of functional products (patent FR2842994A1) [18], and the search for solutions that increase shelf life and convenience (patent US2007243289A1) [19]. These data reflect a growing and sustained interest in innovation in the sourdough sector, particularly in Europe and North America, with significant attention in the Asia–Pacific region (Figure 3). The high number of patents registered in Europe, particularly in Germany, France, and Italy, reflects the need for innovation in countries with a long baking tradition. These innovations aim to improve bakery products’ quality, stability, and organoleptic properties to meet European market demands. For example, patents EP1110458A1 [17] and EP1258526A1 [20] (both developed in Germany) concern innovative processes for sourdough preparation through the combined use of selected yeasts and homo- and hetero-fermentative *Lactobacillus* strains. Another example is the patent WO0010395A1 (Sweden) [21], which describes a process for preparing a liquid sourdough characterized by a long shelf life and high microbial-culture viability. Given its dynamic market orientation toward artisanal processes and healthy products, the United States also exhibits considerable patent activity. The high number of sourdough-related patents in the Asia–Pacific region, particularly in China and Japan, is probably related to the westernization of eating habits and the growing demand for high-quality baked goods in these countries. Finally, patenting in South America, the Middle East, and Africa is limited but growing, indicating an emerging interest in sourdough-derived products.

## 3. Production, Maintenance, and Preservation of Traditional Sourdough (Type I)

**Figure 4:**
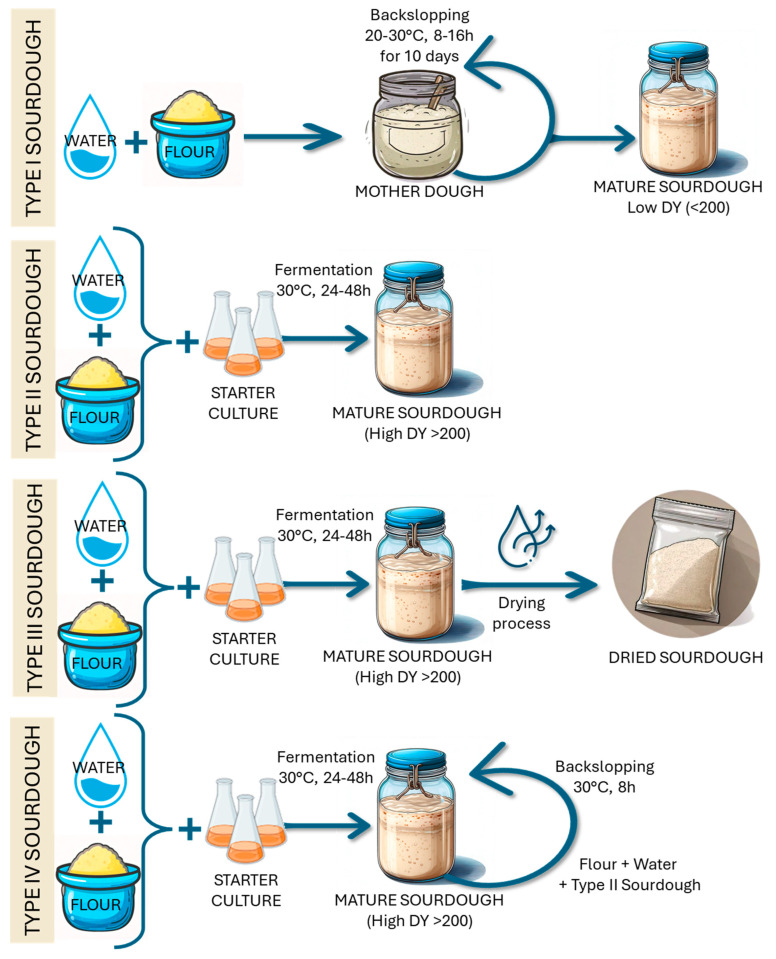
Schematic representation of the different sourdough types (Types I−IV). DY: dough yield.

Sourdough is classified into four types (I, II, III, and IV) based on the microbial inoculation and fermentation approaches (Figure 4) [22]. Each sourdough type represents a different balance between traditional methods and industry-specific standardized protocols, offering various advantages in terms of consistency, efficiency, and customization. This classification system provides a framework for understanding the diverse approaches to sourdough production and their implications for microbial ecology, fermentation dynamics, and final-product characteristics.

Accordingly, each sourdough type harbors diverse microbial communities (Table 1). Type I is the traditional sourdough, which is spontaneously fermented by lactic acid bacteria (LAB) and yeasts present in flour; in the environment; and in fruits, vegetables, or other raw materials that could be mixed with the flour [23,24]. Mature sourdough, which is obtained after multiple backslopping processes with a short-to-moderate fermentation time (6–24 h), reaches a pH of approximately 4.0 and is characterized by a low dough yield (DY < 200). Type I sourdough is usually preserved at 4 °C through continuous backslopping, which requires both time and experience [25]. These factors have contributed to the limited utilization of type I sourdough in industrial production, resulting in a preference for type III sourdough, which benefits from a standardized fermentation environment and the addition of *Saccharomyces cerevisiae* starter strains. This enhances the leavening power and ensures the rapid growth of microbial starters [26] but suppresses the natural diversity of spontaneous yeasts and LAB [27]. Indeed, the addition of *S. cerevisiae* at high cell densities, around 10^7^ CFU/g of dough, shifts the bacteria-to-yeast ratio in favor of increased yeast dominance. *S. cerevisiae* rapidly becomes the predominant species, due to its rapid reproduction, significantly reducing the microbial diversity of non-*Saccharomyces* yeasts [28]. In addition, *S. cerevisiae* consumes flour-derived carbohydrates at a higher rate than heterofermentative lactobacilli, resulting in reduced growth and metabolic activity in this LAB group [29]. The competitive advantage of *S. cerevisiae* is also related to its rapid production of ethanol, which creates a selective environment [9]. Ultimately, the inoculated yeast starter supplants microorganisms originating from flour or water [26].

In this respect, the identification of proper long-term preservation protocols for type I sourdough would represent not only an important economic achievement in the artisanal baking industry but also a challenge for microbial culture collections in terms of new protocols to preserve and safeguard typical traditional baked goods that retain their specificity and uniqueness.

Indeed, the dynamic nature of Type I sourdough microbiota leads to changes in the community composition over time, which is also due to stochastic causes. For instance, the diversity of strains and species that persist in sourdough microbiota, especially LAB, can increase over time [50,51]. Minervini et al. [52] reported intraspecies changes and slightly increased yeast counts 1 year after sampling a specific sourdough starter. Despite this inherent dynamism, standardized processing practices can promote a certain degree of microbial stability. The environment in which backslopping is performed is one of the key factors that contribute to such stability. De Vuyst et al. [53] emphasized the role of the propagation environment in shaping the microbial community. Minervini et al. [54] maintained seven traditional sourdoughs in a laboratory and an artisanal bakery for 80 days through continuous backslopping. The same flour batch and technological parameters were used in both settings. Nonetheless, bacterial diversity analyses—based on both culture-dependent and -independent methods—revealed that in five out of seven cases, the microbial communities diverged depending on the propagation environment. Similarly, Scheirlinck et al. [10] demonstrated that, despite using different flour batches with potentially varying characteristics, bakery-specific strains persisted in doughs over a sampling period of at least 3 years. This long-term persistence is likely due to standardized fermentation parameters and continuous microbial input from the surrounding environment. In this context, the (long-term) preserved microbiota of a mature sourdough may play a role comparable to that of the bakery environment, acting as a microbial reservoir of fully adapted strains. Its use could enable targeted inoculation of the flour–water matrix with specific microbial consortia, thereby facilitating the reconstruction of the original sourdough following disruptive events, such as aggressive sanitation, phage contamination, or abnormal fermentation temperatures. Furthermore, the preservation of isolated microbial cultures and starters represents a critical aspect of modern bread production, especially as new biotechnological approaches are increasingly integrated into baking practices. This includes the use of strains with specific functional properties related to metabolite and enzyme production, contributing to product innovation and consistency [55].

## 4. Methods to Maintain and Preserve Type I Sourdough

### 4.1. Backslopping or Refreshment

The traditional methods of maintaining type I sourdough have played a crucial role in this ancient bread-making technique throughout history. The most common method, backslopping, involves regularly “feeding” the starter with fresh flour and water. This process, also known as “refreshment”, maintains the microbial population in an active state, as it provides microorganisms with fresh nutrients. Feeding frequency depends on various factors, such as environmental temperature, starter maturity, and baking frequency. At room temperature (20−25 °C), most bakers feed their starters once or twice daily, typically in equal parts by weight [6]. It helps maintain an optimal pH level (most commonly range between 3.4 and 4.9) by diluting the acidic fermentation byproducts. Feeding also controls the cellular concentration of microorganisms, thereby preventing alteration of the balance between microbial populations and resource depletion. Repeated backslopping maintains a stable sourdough microbiome at the species level while permitting subtle shifts within species. Through 16S metagenomic analysis, Minervini et al. [52] showed that the bacterial community composition of sourdough was remarkably stable in terms of species when using the same type (but different batches) of flour and the same conditions for sourdough propagation. On the contrary, Taccari et al. [56] observed significant changes in the microbial population of a type I sourdough maintained with daily back-slopping for over 20 days under both laboratory and artisan bakery conditions. Particularly, microbial species typically found in fresh sourdough (*Lactococcus lactis* and *Leuconostoc holzapfelii/citreum* for bacteria, and *Candida silvae* and *Wickerhamomyces anomalus* for yeasts) were progressively replaced by species better adapted to the sourdough ecosystem (*Lactobacillus brevis*, *L. alimentarius, L. paralimentarius,* and *S. cerevisiae*). When sourdoughs propagated with the same flour were compared between laboratory and artisanal bakery settings, both the substrate and the propagation environment were found to influence microbial diversity. Vogelmann and Hertel [57], by varying dough percentages (5−20%), refreshment times (12−24 h), and temperature ranges (20−30 °C and 35−40 °C) during backslopping, observed variations in the initial pH and growth environment, while the microbial association between *F. sanfranciscensis* and *K. humilis* remained stable. Vrancken et al. [58] observed that 10 backslopping cycles (every 24 h) at 23 °C resulted in the dominance of *Leuconostoc citreum*, whereas *L. fermentum* was the dominant species at 30 °C and 37 °C. Longer backslopping intervals (every 48 h at 30 °C) resulted in the co-dominance of *L. fermentum* and *L. plantarum*. This demonstrates the dynamism of the sourdough microbiota and the stressful effect of backslopping temperature on microbial taxonomic composition. Van Kerrebroeck et al. [59] demonstrated that a process comprising 9 backslopping steps at a stable pH of 4.0 increased the lactic acid and ethanol concentrations. These metabolites were related to the positive effect of backslopping on LAB growth (*L. fermentum* and *L. plantarum*, *L. pentosus*, and *L. paraplantarum*) and its negative effect on yeast growth (only *C. glabrata* was able to persist). These studies indicate that factors such as flour composition, pH, and temperature modulate microbial succession and metabolite profiles. On the positive side, this variability allows bakers to customize recipes and techniques to achieve specific bread characteristics. However, there are also disadvantages, including the time, effort, and expertise required for the maintenance of the sourdough, or unpredictable negative outcomes such as contamination from unwanted bacteria or filamentous fungi [60].

### 4.2. Preservation of Sourdough Microbiomes at Low Temperatures

Considering the issues related to continuous backslopping, different approaches based on the use of low temperatures have been evaluated to preserve sourdough microbiota. Siepmann et al. [22] demonstrated that sourdough refrigeration at 4 °C extends the interval between feedings to 1−2 weeks while maintaining viability. However, regular backslopping is necessary because the refrigeration temperature of 4 °C does not protect the sourdough from filamentous fungi contamination [61]. Additionally, the starter usually requires a few feeding cycles at room temperature to regain full activity after removal from the refrigerator. Finally, the microbial community composition of sourdough can be affected at the species level during refrigeration at 4 °C. For example, *F. sanfranciscensis* maintains its metabolic activity at 4 °C, albeit at a reduced rate [62]. Some autochthonous flour yeasts, such as *K. exigua*, have shown better cold tolerance than *S. cerevisiae* [63]. Lattanzi et al. [64] showed that the core microbial species of sourdough samples were maintained after 30, 60, and 90 days of storage at 4 °C, with *L. sanfranciscensis* A4 and *L. plantarum* DB200 consistently detected throughout the storage period. However, DGGE analysis highlighted changes in the occurrence of less abundant species, including the appearance of lactic acid contaminants, which may affect sourdough performance upon reactivation.

**Figure 5:**
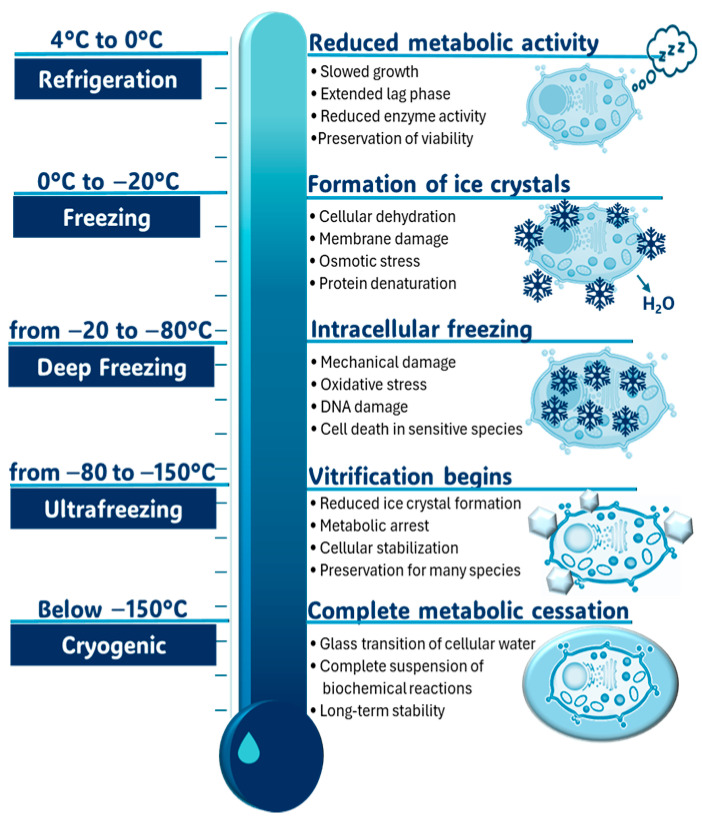
Effects of low temperature on microbial cells.

Freezing at −20 °C and −80 °C has the potential to preserve sourdough microbiota for long-term (even years). However, freezing has significant negative effects on microbial survival [64]. Reale et al. [65] showed that freezing sourdoughs reduced LAB and yeasts’ viable cells by approximately 4 logarithmic values. Accordingly, Chen et al. [66] measured LAB counts in sourdough made with *L. plantarum* M616 and noted a decline from 7.5 to 6.0 log CFU/g over 60 days at approximately −18 °C. Lattanzi et al. [61] observed more-than-4-log reductions in yeast cell viability in three sourdoughs after 7 days of freeze-storage and suggested that freezing and subsequent thawing severely injured yeast cells. Additionally, DGGE analysis showed that LAB species were preserved during freezing for up to 90 days, with *L. sanfranciscensis* A4 and *L. plantarum* DB200 consistently detected throughout the storage period. Li et al. [67] observed that after storage at −20 °C, the abundance of *Acetobacter* increased, whereas that of *Lactobacillus* decreased in Jiaozi (JZ) sourdough starters. Freezing was also associated with an increased relative abundance of *Saccharomyces*. Metagenomic sequencing indicated that JZ microbial communities were able to adapt to freezing stress by up-regulating metabolic pathways associated with oxidative phosphorylation, glutamate biosynthesis, and unsaturated fatty acid production. This adaptive response translated into enhanced fermentative characteristics after thawing, with high yeast vitality and fermentative activity. Minervini et al. [68] found that sourdoughs with a LAB-to-yeast ratio of 100:1 or higher exhibited better stability during storage than those with lower ratios. The impact of freezing can vary depending on the strains present in the sourdough and factors such as the dough composition and freezing parameters. The higher decline in microbial viability during freezing at −20 °C, compared to −80 °C, is likely due to the formation of larger ice crystals at higher temperatures [69] (Figure 5).

Thus, one of the main objectives for the long-term storage of bacterial and fungal strains at low temperatures is to identify the most effective cryoprotectants (CPAs). This is particularly important in the cryopreservation of microbial communities because the process inherently favors the survival of freeze-tolerant microorganisms. Consequently, if not carefully optimized, particularly through the appropriate selection and application of CPAs, the process may impose selective pressures on the microbial community, potentially leading to alterations in its original composition and functional properties [70]. In regard to this matter, several studies have explored the use of CPAs for the cryopreservation of microbiomes from diverse environments; however, only a limited number have specifically examined their effects on sourdough-associated microbial communities. Kerckhof et al. [71] developed a cryopreservation protocol using dimethyl sulfoxide (DMSO) alone or in combination with trehalose and tryptic soy broth (DMSO + TT) for the storage at −80 °C of a methanotrophic co-culture (MOB), an OLAND biofilm, and a human fecal microbiome. Among the CPAs tested, both DMSO and DMSO + TT promoted faster recovery of metabolic activity and preservation of the microbial community structure in the MOB (156% and 96.47%, respectively) and OLAND (94.5% with DMSO + TT) microbiomes, maintaining functional stability even after extended storage. Aguirre et al. [72] reported that glycerol was the most effective CPA for preserving human fecal microbiomes at −80 °C, ensuring the survival of key phyla, such as *Actinobacteria*, *Firmicutes*, *Fusobacteria*, *Verrucomicrobia*, and *Proteobacteria*, while *Bacteroidetes* showed a noticeable decline. Similarly, Yu et al. [73] found comparable efficacy between glycerol and DMSO in preserving a grass-enriched microbial community, although DMSO-preserved samples required longer recovery times to reach pre-storage functionality. In the context of food-associated microbiota, Teotônio et al. [74] assessed the effect of different CPAs in gluten-free frozen-dough systems. They found that a combination of 69% fructo-oligosaccharides and 31% hydrolyzed soy protein provided superior protection for *Saccharomyces* fermentation capacity, suggesting their potential as cryoprotectants in frozen dough formulations. Ongoing research aims to improve the efficiency of CPA, elucidate their mechanisms of action, and expand their applications to microbiomes of interest for the food and agriculture sectors [75].

### 4.3. Freeze-Drying

Freeze-drying is the most important industrial method for storing sourdoughs, as it offers several advantages over conventional drying methods but also presents limitations related to equipment and product formulation [76]. The process involves three main stages: freezing, primary drying (sublimation), and secondary drying (desorption). The desired freezing rate (°C/min), whether performed directly or using external equipment, influences the freezing step, which affects the development of ice crystals and formation of pores in cell membranes [77]. Slow freezing tends to create larger ice crystals on the outside of cells, resulting in crystalline regions; however, it can also damage the lipid structures of cell membranes, ultimately reducing microbial viability. Smaller intracellular, amorphous ice crystals are formed during rapid freezing, and the cells are preserved [78]. After months or even years, freeze-drying causes only minor reductions in LAB and yeast populations within the sourdough microbiota. However, there were instances where freeze-drying did not achieve the desired microbial viability [79]. For instance, Reale et al. [65] showed that a type I sourdough with initial LAB and yeast populations of 9.17 and 7.53 log CFU/g, respectively, experienced a decline of 3 log CFU/g following six months of storage at room temperature after freeze-drying. Additionally, Caglar et al. [80] measured a reduction of 1.7 log CFU/g for both LAB and yeast after freeze-drying a sourdough sample. The decrease in microbial viability may be attributed to the prolonged exposure of the sample in the equipment (48 and 60 h) without protective agents, which can lead to microbial inactivation due to cryogenic damage [81]. Therefore, freeze-drying usually requires the use of a lyoprotectant to prevent cell damage during freezing. As with CPAs, the selection of the optimal lyoprotectant remains an active area of research that requires further investigation across diverse microbial groups. Simple sugars (glucose, fructose, lactose, mannose, sucrose, sorbitol, adonitol, and trehalose) and more complex matrices, such as skim milk, arabic gum, monosodium glutamate, starch, and oligosaccharides, have been investigated for their ability to protect bacterial cells during the drying process. Gul et al. [82] achieved a survival rate of over 94% for *Levilactobacillus brevis* ED25 after 180 days at 4 °C using a mixture of 17.28% skim milk, 2.12% lactose, and 10% sucrose during freeze-drying. As a reference, the same strain in the same conditions, but without lyoprotection, reached a survival rate of 72%. The protective effect was primarily attributed to skim milk, which prevented ice-crystal formation, while lactose improved stability and reduced cell damage. Stefanello et al. [83] studied the preservation of *L. fermentum* and *W. anomalus* isolated from sourdough after freeze-drying with four lyoprotectants (10% sucrose solution; 5% trehalose solution; 10% skim milk solution; and a stabilizing solution containing 5% sodium L-glutamate monohydrate p.a. and 10% skim milk powder). The most effective lyoprotectants for both microorganisms were skim milk and skim milk with glutamate, allowing 74%–87% recovery of *L. fermentum* and 80% of *W. anomalus* viability after lyophilization and storage at room temperature for 1 year. De Valdez and Diekmann [84] reported that glutamate provided the highest cell survival rate (80%) for *L. reuteri* after freeze-drying, with counts remaining above 8.0 log CFU/g after six months at various storage temperatures (−20, 4, and 25 °C). The lyoprotectant concentration to be added to cell suspensions is another area worth considering. Stefanello et al. [85] showed that the protective effect was proportional to the concentration of added protectants: the higher percentage of the added protective agent, the greater the cell viability. Gul et al. [82] showed that when the surface of sourdough samples was not completely covered by the protective agent during the drying process, irregular water loss, increased gas permeability, and lower cell protection occurred. As there are no optimal methods that can be equally applied for all types of microorganisms, more work needs to be performed in this area.

### 4.4. Oven Drying

Oven drying is a common method for producing dried sourdoughs that can be directly incorporated into bread-making, improving various bread properties, such as specific volume, pH, acidity, antioxidant activity, and sensory characteristics [49]. However, Reale et al. [65] suggested that the thermal damage during oven drying can significantly reduce viability of the sourdough microbiota, impairing fermentation and leavening. Accordingly, Lattanzi et al. [61] showed that after oven drying at 40° C for 20 h, yeast cells did not restore their full functionality following reactivation. In particular, after 3 months of storage, the yeast viability decreased from 7 to 5 log CFU/g, leading to insufficient leavening capacity when the dried sourdough was used for baking. Ertop and Coşkun [86] obtained similar results: spontaneously fermented chickpea-based sourdough was oven-dried at 35 °C until a residual moisture of 4–5% was reached. While the number of LAB and yeast before drying was 9.47 and 5.3 log CFU/g, respectively, they decreased to 8.25 and 1.3 log CFU/g after drying. According to these results, the drying process negatively affected yeast viability, necessitating the addition of yeast starter in subsequent baking trials. In addition, surviving microorganisms may have diminished metabolic activity, affecting gas production, which is essential for dough rising after rehydration. Finally, the loss of volatile flavor and aroma compounds due to heat can damage the characteristic taste of sourdough bread, reduce its volume, and result in an undesirable texture. Excessive drying can result in products that are too dry or brittle, compromising storage stability and overall quality. In summary, the careful control of drying conditions is crucial for minimizing negative impacts on microbial viability, functional properties, and overall quality of sourdough.

### 4.5. Spray-Drying

Spray-drying is an efficient atomization method that involves multiple stages. In this process, which is frequently used in industry, a product is atomized into droplets within a chamber where the circulation of hot air (150−200 °C) facilitates heat transfer and solvent evaporation, ultimately solidifying the liquid particles [87]. Although spray-drying is cheaper and more energy-efficient than freeze-drying, it is not commercially widespread due to problems such as low cell-survival rates, limited storage stability, and rehydration difficulties [88]. The industrial production of dried sourdoughs primarily uses drum-drying and spray-drying techniques, with rye and wheat flour as common substrates [89]. Although these processes achieve a reasonable cell survival rate, the drying conditions and equipment configurations should be carefully studied [90]. The spray-drying time and the air-outlet temperature are critical adjustable parameters. Ertop et al. [49] suggested that exposing sourdough to spray-drying for 2–3 s can help maintain the white color of the final product. In both the studies by Caglar et al. [80] and Tafti et al. [48], an outlet temperature of 90 °C resulted in a 4 log CFU/g viability loss for sourdough yeasts and bacteria. Mohd Roby et al. [91] utilized an air-outlet temperature of 100 °C but incorporated 7% arabic gum as a protective agent during the drying of sourdough and obtained a survival percentage of 90.27% and 89.52% for LAB and yeasts, respectively.

## 5. “De Novo” Design of Reconstructed Microbial Communities as a Sourdough Reproduction and Preservation Method

In addition to preserving whole sourdoughs and their associated microbial communities, microbial culture collections can also store and supply individual strains for the development of “artificial”, “synthetic”, or “engineered” microbial consortia [70]. These synthetic consortia represent a compromise between the need to preserve the complexity of natural microbiomes and the practical challenges of their preservation and distribution. They offer a controlled and reproducible approximation of a complex microbiome, enabling standardized studies and industrial applications. The inclusion of microbial strains isolated from a specific sourdough would allow for the preservation of the unique identity of such a food matrix. Identifying the key microbial species or strains that best represent the entire sourdough community is a critical aspect in the development of synthetic consortia. Particularly, a combination of dominant, subdominant, and satellite species is essential for a sourdough synthetic consortium to ensure resilience and optimal performance. This diversity should provide gene and transcript redundancy, which is vital for preserving metabolic functions despite consistent biotic pressures and spatiotemporal changes, which can lead to community instability [52]. Arora et al. [8] identified up to 59 culturable bacterial genera in sourdough, with *Lactobacillus* being the most abundant, represented by 82 distinct species that are primarily nomadic and heterofermentative. The inspection of metagenomes and transcriptomes by Calabrese et al. [92] revealed the complexity of the key sourdough microbiome taxa. In eight spontaneous sourdoughs, the dominant species included bacteria such as *F. sanfranciscensis*, *L. fermentum*, *L. plantarum*, *L. citreum*, and *S. cerevisiae*. Subdominant (*L. paracasei*, *L. rhamnosus*, *F. rossiae*, *P. pentosaceus*, *L. mesenteroides*, *L. pseudomesenteroides*, *L. lactis*, *E. faecium*, and *Erwinia persicina*) and satellite (such as *Staphylococcus epidermidis*) species were found to vary in different sourdoughs. These species are characterized by a low prevalence (below 5% relative abundance in at least one sourdough) but are considered to contribute to the community’s functional redundancy and stability, especially under fluctuating environmental conditions. The selective inclusion of these species in custom consortia allowed the researchers to evaluate their specific contributions to sourdough performance and successfully reconstruct sourdough synthetic microbial communities. The synthetic community included seven species: *S. cerevisiae*, *P. kudriavzevii*, *L. plantarum*, *L. fermentum*, *F. rossiae*, *P. pentosaceus*, and *S. epidermidis*. Overall, the study emphasizes the importance of microbial diversity and species interactions in maintaining the functionality and stability of sourdough metacommunities. Particularly, over 30 days of propagation, the reconstructed synthetic sourdough community maintained a stable volatilome profile [92]. This suggests that a consistent flavor can be reproduced from preserved strains, at least under controlled conditions. However, simplifying a sourdough ecosystem in this way does not capture the full complexity of type I sourdoughs, especially for the more subtle or specific aspects of flavor. A recent study by Rappaport et al. [93] more closely evaluated the role of acetic acid bacteria in shaping sourdough characteristics. Interestingly, they found that even strains with over 97% average nucleotide identity could lead to meaningful differences in microbial function. These differences influenced the bread’s sensory qualities. This finding underscores the importance of microbial diversity in the development of flavor. Indeed, the community structure affects not only fermentation but also the production of volatile precursors in reactions like lipid oxidation and the Maillard reaction, both of which play major roles in developing the aroma and taste of the final bread [94].

## 6. Microbial Culture Collections and Microbial Biological Resource Centers

Microbial culture collections (MCCs) are essential ex situ repositories for the preservation of microbial biodiversity and provide authenticated reference material for biotechnology research and innovation [95]. According to the World Data Centre for Microorganisms [96], there are currently 873 culture collections registered in 80 countries and regions, accounting for a total of 4,238,072 preserved microorganisms. By expanding the scope of their activities and adopting enhanced operational practices and regulatory frameworks, many MCCs have evolved into more complex organizations, i.e., microbial biological resource centers (mBRCs) [97]. These serve as essential biotechnology infrastructures, functioning as repositories for living cells, genomes, and hereditary information while maintaining rigorous quality standards. The regulatory frameworks implemented in mBRCs comprise quality management systems such as the ISO 9001 [98] certification and ISO 17025 [99] accreditation. The recent ISO 20387 [100] standard specifically addresses biobanking requirements for biological material acquisition, storage, and distribution [101]. Standards applicable to mBRCs provide the necessary guidelines to ensure consistency, efficiency, and responsiveness to user needs [102]. In addition to internal quality management systems, MCC and mBRCs must comply with international regulatory frameworks regarding the use and sharing of genetic resources. The Nagoya Protocol [103], which was adopted in 2014 under the “Convention on Biological Diversity”, establishes fair and equitable benefit-sharing arising from the utilization of genetic resources [104,105]. It ensures that countries providing genetic resources, including their associated traditional knowledge, retain sovereign rights over their use and receive fair compensation through access and benefit-sharing (ABS) measures [106]. Regulation (EU) No. 511/2014 introduces a common approach to regulatory control in Europe and requires due diligence to respect compliance practices [107]. Another essential aspect covered by MCC and mBRCS is the management of data associated with microbial resources. Pure cultures of microbial strains are typically assigned unique identifiers and are recorded in a database alongside descriptive metadata. These may include information about the source of isolation (e.g., substrate, date, geographic location, depositor, and any ancestral strains), cultivation history, phenotypic traits (e.g., morphology, metabolic activity, or antibiotic resistance), molecular data (including genotypes, and genomic or plasmid sequences), potential applications, the relevant literature, and optimal growth conditions. The extent of publicly accessible information varies among mBRCs, with many providing full or partial access through online catalogues. To mitigate excessive fragmentation in access control to genetic resources, as well as in data management and sharing, the World Data Centre for Microorganisms, in collaboration with the World Federation for Culture Collections, coordinates public service collections by developing tools such as the “Global Catalogue of Microorganisms” and other key resources [96,108]. Regional cooperation is promoted through specialized organizations, including the European Culture Collections’ Organization [109], the Asian Consortium for the Conservation and Sustainable Use of Microbial Resources [110], the United States Culture Collections Network [111], and the Latin American Federation for Culture Collections [112]. Recently, there has been a higher level of integration between the microbial and human sectors, as demonstrated by the Lusophone Network of Biobanks and Biological Collections, which strengthens collaboration and resource sharing [113]. Specific catalogues, such as the “Italian Culture Collections Catalogue”, demonstrate a balance between centralized coordination and flexibility at the national level [114]. Recent trends in data management protocols have focused on the implementation of FAIR principles (findable, accessible, interoperable, and reusable) to ensure standardized practices in data collection, storage, and sharing across MCC and mBRCs. According to MIRRI-ERIC, the organization of databases related to microbial resources should accommodate two main strategies: interoperability and unified access. Casaregola et al. [115] highlighted that the adoption of common ontologies that support data standardization and allow for an effective integration is crucial for implementing interoperability.

A unique example of a sourdough collection is the “Sourdough Library” [116], which was opened in 2013 by Puratos in Belgium. Each new sourdough sample admitted to the collection is collected into three jars and immediately stored at 4 °C. One aliquot is analyzed in the Puratos chemical laboratory; the second is sent to the Free University of Bozen/Bolzano for microbial strain isolation; and the third is maintained at 4 °C, with monthly backslopping, serving as a backup in case the original bakery requires restoration. The Sourdough Library’s long-term preservation strategy follows a “single-strain” approach, focusing on the isolation and cryopreservation of individual strains as pure cultures at −80 °C [117]. To date, the collection includes over 900 strains of wild yeasts and lactic acid bacteria, representing a valuable resource for the conservation of microbial diversity in traditional sourdoughs.

## 7. Current and Future Challenges of MCCs and mBRCs

In the era of microbiome research, mBRCs and MCCs are increasingly required to adopt specific preservation methods that guarantee the stability and quality of the stored microbiome. They should also implement systems for cataloguing microbiome-associated data while complying with national and international regulatory frameworks. The traditional operating models of MCCs and mBRCs are based on the propagation, preservation, documentation, and sale of individual microorganisms [118]. However, this approach has limitations regarding microbiomes, such as those associated with sourdough samples. Microbiomes’ functions are typically performed not by individual species but by metacommunities. The structure, functioning, resilience, and stability of these metacommunities result from a dynamic interplay between natural selection and random events in a way that is still poorly understood [119]. Such functions and activities cannot be performed by a single strain or a couple of isolates separated from the microbiota. Recognizing the limitations of traditional preservation approaches, several research projects and infrastructures have been recently developed to identify suitable methods and protocols to extend classical strain collections to complex microbiomes, such as the BBMRI-ERIC Microbiome Expert Group (2019–ongoing), the MICROBE–MICRObiome Biobanking (RI) Enabler (Horizon Europe 2023–2027) [120], the IS_MIRRI21/MIRRI-ERIC (H2020 2020–2024) [121], etc. Additionally, the development of appropriate sample-preservation methods should consider the need for accurate microbiome profiling at the molecular level to ensure the preservation of microbiome quality and integrity. In this regard, a recent study by Smenderovac et al. [122] evaluated the impact of six preservation methods—CD1 solution, 95% ethanol, Dry & Dry silica gel packs, RNAlater, LifeGuard, and freezing at −20 °C—on DNA concentration and quality, as well as on the taxonomic composition of bacterial and fungal communities. Although storage at −20 °C was recognized as the most effective approach for maintaining high-quality DNA, drying also enabled room-temperature storage of samples suitable for downstream DNA-based microbiome analyses [123]. By safeguarding microbial biodiversity and related data, mBRCs should ensure that valuable resources, such as the microbiomes of traditional fermented foods, will be accessible to the scientific community and preserved for future generations [124], contributing to the preservation of the national gastronomic heritage and its scientific valorization [125]. As climate change threatens agricultural biodiversity, preserving diverse sourdough cultures could also guarantee food security.

Regarding compliance with regulatory frameworks governing the use and sharing of microbial resources, Document 2021/C 13/01, published in the *Official Journal of the European Union* by the European Commission, clarifies the applicability of Regulation (EU) No. 511/2014 to microbial communities from various origins [126]. While human microbiota studies in situ or those examining unique compositions within an individual are excluded, activities involving the isolation and analysis of microbial taxa from human microbiota fall under this regulation. Similarly, research and development activities involving microbial strains from plants, animals, or environmental samples are subject to regulation if they are associated with genetic resources from countries with ABS measures under the Nagoya Protocol, although it does not explicitly indicate how to deal with microbiomes. This includes microbiomes of agri-food origin, such as those in fermented foods or agricultural environments. Users must negotiate “prior informed consent” and “mutually agreed terms” when the microbiome is linked to resources covered by ABS laws. Thus, only microbiomes of unknown origin or accidentally introduced contaminants are excluded. Proper documentation, clear microbiome origin tracing, and adherence to ABS protocols are strongly recommended to ensure compliance with the above-mentioned regulations.

Finally, all data harmonization initiatives, which are now applied to single pure strains, need to be implemented and uploaded to catalog and share microbiome-associated data. The lack of interoperability between databases is a major challenge, preventing complex searches between databases of different microbial collections and between them and metagenomics information systems. Furthermore, a clear link to involved microbial resources and the detailing of single strains, as present in mBRC databases, is missing. Standards for minimum metadata applicable to food microbiome databases have been developed to facilitate comparative analyses across microbiome studies, and their harmonization is essential to enable comparative analyses across different studies. The Minimum Information about Any Sequence (MIxS) guidelines of the Genomic Standards Consortium (GSC) presented by Yilmaz et al. [127] provide a basic framework. Parente et al. [128] highlighted the importance of specific parameters for food microbiomes and presented the structure of the database for their software FoodMicrobionet (v5). Both MIxS and the FoodMicrobionet data structure incorporate some information on microbial strains identified in the microbiota, but a description of their characteristics is not included. Furthermore, it is emphasized that microbiome data, as with pure culture strains, must include taxonomic details, origin, physiological characteristics, and bibliographic references, which are considered essential for effective research and data validation. Stackebrandt et al. [124] also mention the commitment to provide high-quality data according to agreed standards, as part of the MIRRI-ERIC Partner Charter, so that all culture collections participating in the infrastructure may provide coherent and homogeneous quality data and can provide further insights into relevant physio-chemical properties of strains of potential major interest for metagenomics. Metadata based on interoperability and integration should be implemented in food microbiomes as well, confirming the importance of a standardized but comprehensive approach to the cataloguing and organization of microbiome information to ensure that it is findable, accessible, interoperable, and reusable (FAIR principles), as outlined in the latest trends in microbial data management for MCCs and mBRCs.

## 8. Conclusions

Although the advantages of preserving sourdough microbiomes are evident, several challenges that require technical advances and updated regulatory and legal frameworks must be considered. First, no standardized protocols are currently available for the long-term preservation of sourdough microbiomes, particularly type I sourdough, a dynamic microbial niche that is constantly evolving. Therefore, proper storage methods are required to prevent significant changes in the taxonomic composition and functionality of the sourdough microbiome. Second, ethical considerations exist regarding the ownership and use of stored sourdough microbiomes, especially those with cultural significance. Well-defined access and benefit-sharing regulations are required to ensure fair and sustainable use. Documenting and safeguarding fermented-food biodiversity and traditional practices is also essential for future food innovation and research. In this context, specialized microbial biological resource centers are expected to play a key role in developing and implementing standardized protocols to document and preserve the microbiota of endangered fermented foods, thereby ensuring future generations’ access to these invaluable resources.

## Figures and Tables

**Table 1:** Overview of the main LAB and yeast species identified in the different types of sourdough.

Sourdough Type	Lactic Acid Bacteria (LAB)	Yeast	References
Type I	*Lactobacillus sanfranciscensis*, *Lactobacillus pontis.*	*S. cerevisiae*, *Kazachstania humilis.*	[30]
	*Levilactobacillus brevis*, *Lactiplantibacillus plantarum*, *Lacticaseibavillus rhamnosus.*	*Wickerhamomyces anomalus*, *S. cerevisiae*, *Torulaspora delbruekii*, *Pichia kluyveri*, *Candida boidinii*, *Candida diddensiae.*	[31]
	*L. brevis*, *Lactobacillus alimentarius*, *Lactobacillus pentosus.*	*S. cerevisiae*, *K. humilis.*	[32]
	*Fructilactobacillus sanfranciscensis*, *L. plantarum*, *Lactobacillus kimchi*, *Lactobacillus sakei*, *Lactobacillus hamesii*, *L. pentosus.*	*Kazachstania bulderi*, *Candida humilis*, *Kazachstania unispora*, *T. delbruekii*, *Rhodotorula mucillaginosa*, *Candida carpophila*, *S. cerevisiae*, *Hyphopichia pseudoburtonii.*	[33]
	*L. plantarum*, *L. sanfranciscensis*, *Lactobacillus spicheri*, *Lactobacillus rossiae*, *Lactobacillus namurensis*, *Lactobacillus zymae*, *Lactobacillus casei*, *Lactobacillus mindensis*, *Lactobacillus acetotolerans*, *Lactobacillus farciminis*, *Lactobacillus paralimentarius*, *Pediococcus pentosaceus*, *Enterococcus durans*, *Enterococcus faecium*, *Leuconostoc mesenteroides*, *Weissella confuse.*	*S. cerevisiae*, *Pichia guiliermondii*, *T. delbrueckii*, *Candida parapsilosis*, *Candida pararugosa.*	[34]
	*L. sanfranciscensis*, *W. cibaria*, *Lactobacillus fermentum*, *L. plantarum*, *L. pontis*, *L. paralimentarius.*	*S. cerevisiae*, *C. humilis*, *W. anomalus.*	[35]
	*Weissella viridescens*, *P. pentosaceus*, *Pediococcus acidilactici*, *L. brevis*, *Lactobacillus parabuchneri.*	*S. cerevisiae*, *Pichia membranifaciens.*	[36]
	*F. sanfranciscensis*, *Companilactobacillus paralimentarius*, *L. brevis.*	*S. cerevisiae*, *K. humilis*, *K. bulderi.*	[37]
	*L. curvatus*, *F. sanfranciscensis*, *Leuconostoc citreum*, *Leuconostoc mesenteroides*, *Leuconostoc pseudomesenteroides*, *P. pentosaceus*, *Lactobacillus acidifarinae.*	*C. humilis*, *T. delbrueckii*, *S. cerevisiae*, *Kluyveromyces marxianus.*	[38]
	*F. sanfranciscensis.*	*Candida milleri*, *S. cerevisiae.*	[39]
	*Lactobacillus fructivorans*, *L. plantarum*, *Lactobacillus reuteri*, *Lactobacillus delbrueckii*, *Weisella spp.*	*C. humilis*, *S. cerevisiae*, *P. kudriavzevii.*	[40]
	*F. sanfranciscensis*, *W. cibaria*, *L. plantarum*, *L. reuteri*, *L. pontis.*	*S. cerevisiae*, *Kazachstania exigua.*	[41]
Type II	*Leuconostoc citreum*, *Lactococcus lactis*, *W. confusa*, *W. cibaria*, *L. plantarum*, *Lactobacillus paraplantarum*, *L. brevis.*	*W. anomalus*, *Kazachstania unispora*, *S. cerevisiae.*	[42]
	*L. curvatus*, *P. pentosaceus*, *L. brevis*, *L. plantarum*, *L. mesenteroides*, *L. rossiae.*	*S. cerevisiae.*	[43]
	*L. plantarum*, *L. reuteri*, *L. delbrueckii.*	*S. cerevisiae*, *W. anomalus*, *Saccharomyces bayanus*, *T. delbrueckii.*	[40]
	*L. fermentum*, *L. plantarum*, *L. brevis*, *W. confusa*, *P. pentosaceus.*	*S. cerevisiae.*	[44]
	*L. plantarum*, *L. fermentum*, *L. reuteri*, *Lactobacillus acidophilus*, *Lactobacillus johnsonii*, *Lactobacillus farciminis*, *L. delbrueckii*, *Lactobacillus amylovorus*, *L. fermentum*, *L. sanfranciscensis*, *L. brevis*, *L. pontis*, *Lactobacillus panis*, *Lactobacillus frumenti*, *Weissella spp.*	*S. cerevisiae (added).*	[45]
Type III	*P. pentosacesus.*	*S. cerevisiae (added).*	[46]
	*L.brevis*, *L. fermentum*, *L. frumenti*, *L. pontis*, *L. panis*, *L. reuteri*, *L. sanfranciscensis*, *W. confusa*, *L. acidophilus*, *L. delbrueckii*, *L. amylovorus(rye)*, *L. farciminis*, *L. johnsonii.*	*S. cerevisiae (added).*	[47]
	*L. paralimentarius.*	*S. cerevisiae (added).*	[48]
	*L. delbrueckii*, *L. brevis*, *L. plantarum*		[49]

## Data Availability

No new data were created in this study. Data sharing is not applicable to this article.

## Abbreviations

The following abbreviations are used in this manuscript:

mBRC	Microbial biological resource center
LAB	Lactic acid bacteria
USD	United States Dollar
CPC	Cooperative Patent Classification
MCC	Microbial culture collection
WDCM	World Data Centre for Microorganisms
ABS	Access and benefit-sharing
MIxS	Minimum Information about Any Sequence
GSC	Genomic Standards Consortium
FAIR	Findable, accessible, interoperable, and reusable
JZ	Jiaozi starter
CPAs	Cryoprotectants
